# PB.13: Comparison between analogue and digital mammography: a reader's perspective

**DOI:** 10.1186/bcr3513

**Published:** 2013-11-08

**Authors:** H Gay, R Pietrosanu, S George, D Tzias, R Mehta, C Patel, S Heller, L Wilkinson

**Affiliations:** 1 St George's Hospital NHS Trust, London, UK

## Introduction

In comparison with analogue film, digital mammography provides images with more contrast and allows image manipulation. This study compares features identified on digital and analogue mammograms.

## Methods

During the transition to digital mammography between June and August 2011, women who had received analogue screening mammography and required further views at assessment had repeat digital images. Eight readers reviewed digital and analogue images separately, marking all features that caught their attention on a pro forma. Readers commented on breast density and characterised all features using BiRads descriptors.

## Results

Twenty-three women were recalled for soft tissue lesions (six malignancies) and 13 for calcifications (one malignancy). Fifty analogue/digital single-view mammogram pairs were obtained, 11 included histologically malignant abnormalities. Breast density scores were lower on digital than analogue (*P *< 0.001). There was no significant difference in the descriptions of calcifications. More inconsequential soft tissue features were described on analogue. Soft tissue features tended to be scored as less conspicuous on analogue than digital images. There was no significant difference in the description of 5/6 soft tissue cancers, but one cancer was seen by five readers on analogue, and only two on digital mammogram. See Table 1.

**Table 1:** Aggregate features identified (number of times features were marked)

	Analogue	Digital
Soft tissue	398	319
Calcifications	178	173

## Conclusion

This study showed that readers reported breasts as less dense and identified fewer distracting soft tissue lesions on digital mammography but there was no difference in the reporting of calcifications. One of six cancers was under-reported on digital mammography.

